# *Sargassum horneri* Fucoidan Suppresses the Early-Stage Amyloid Fibrillation of Insulin

**DOI:** 10.3390/ijms27219516

**Published:** 2026-10-24

**Authors:** Noriko Amishiro, Asuka Miwa, Shiho Kariya, Yuka Ito, Yukihiro Yasaski, Minoru Funakoshi, Takashi Ohshiro, Hisashi Yagi

**Affiliations:** 1 Department of Engineering, Graduate School of Sustainability Science, Tottori University, 4-101 Koyamacho-minami, Tottori-City 680-8552, Tottori Prefecture, Japan; am.noriko322@gmail.com (N.A.); shiho.kariya0294@gmail.com (S.K.); 2 Department of Chemistry and Biotechnology, Graduate School of Engineering, Tottori University, 4-101 Koyamacho-minami, Tottori-City 680-8552, Tottori Prefecture, Japan; miwachan.pooh-chibi@au.com (A.M.); yuka.ito8812@gmail.com (Y.I.); gsnhjpandawest@gmail.com (Y.Y.); 3 Marine Products KIMURAYA Co., Ltd., 3307 Wataricho, Sakaiminato-City 684-0072, Tottori Prefecture, Japan; funakoshi@fucoidan1.jp; 4 Department of Chemistry and Biotechnology, Faculty of Engineering, Tottori University, 4-101 Koyamacho-minami, Tottori-City 680-8552, Tottori Prefecture, Japan; ohshiro@tottori-u.ac.jp; 5 Center for Research on Green Sustainable Chemistry, Tottori University, 4-101 Koyamacho-minami, Tottori-City 680-8552, Tottori Prefecture, Japan; 6 Research Center for Utilization of Unused Biological Resources, Tottori University, 4-101 Koyamacho-minami, Tottori-City 680-8550, Tottori Prefecture, Japan

**Keywords:** amyloid fibrils, insulin, Sargassum horneri, fucoidan, sulfated polysaccharide, fibrillation inhibition

## Abstract

Amyloid fibril formation is implicated in numerous protein misfolding disorders. Fucoidan, a sulfated polysaccharide primarily derived from brown algae, exhibits diverse biological activities. In this study, we investigated the inhibitory effect of *Sargassum horneri* fucoidan (S-fucoidan) on insulin amyloid fibrillation in vitro. Initial screening of fucoidans derived from *Cladosiphon okamuranus*, *Kjellmaniella crassifolia*, and *S. horneri* identified S-fucoidan as the most effective inhibitor of insulin fibrillation. Therefore, we characterized the inhibitory effect of S-fucoidan using Thioflavin T fluorescence, transmission electron microscopy, circular dichroism spectroscopy, size-exclusion chromatography, and cell viability assays. Both S-fucoidan and low-molecular-weight S-fucoidan inhibited fibrillation in a concentration-dependent manner. When present from the onset of the reaction, S-fucoidan prevented detectable fibril formation, preserved the initial secondary structure of insulin, and maintained insulin in a monomeric or near-monomeric state. In contrast, addition during nucleation or elongation did not effectively halt ongoing fibrillation. These findings indicate that S-fucoidan predominantly suppresses the early stage of insulin amyloid fibrillation and provide a basis for investigating the molecular mechanisms underlying its anti-fibrillation activity.

## 1. Introduction

Abnormal protein aggregation is implicated in a wide range of human diseases [1,2,3]. Amyloidosis is characterized by the deposition of proteins into highly ordered fibrillar structures that exhibit amyloid-specific histological characteristics [4,5]. Similar protein aggregation mechanisms are fundamental to neurodegenerative disorders such as synucleinopathies and tauopathies [6,7,8,9]. Despite variations in precursor proteins and affected tissues across diseases, amyloid formation typically involves the transformation of soluble protein species into β-sheet-rich assemblies through nucleation and fibril elongation processes. Therefore, understanding the factors that influence these fundamental steps is crucial for elucidating amyloid formation and developing strategies to manage pathological protein aggregation [10,11,12,13,14].

Amyloid fibrillation has been extensively studied using both disease-associated proteins and experimentally manageable protein models. Many proteins can form amyloid-like fibrils under suitable conditions, regardless of their association with diseases [15]. Partially unfolded conformations can reveal aggregation-prone regions, facilitating intermolecular interactions [16,17]. Amyloid formation is generally characterized by nucleation and elongation processes, whereas concepts such as supersaturation and liquid-liquid phase separation have further broadened the current perspectives on protein aggregation [18,19,20,21,22]. Thus, in vitro model systems are valuable for analyzing the physicochemical factors that promote or inhibit protein aggregation in vivo.

A range of agents, including small molecules, peptides, antibodies, and natural products, modulate amyloid formation [23,24,25,26,27,28,29]. These agents act at various stages of the aggregation pathway. Certain compounds inhibit initial nucleation or monomer association, whereas others bind to the surface or ends of fibrils to block elongation or disassemble the existing fibrils [24,25,30,31]. It is crucial to distinguish these modes of action because the suppression of fibril formation from the onset of a reaction is mechanistically distinct from stopping the elongation or disassembling of mature fibrils.

Marine algae are a source of diverse bioactive compounds, including polyphenols and structurally unique polysaccharides [32,33,34,35,36,37,38,39]. Brown algal polyphenols have been identified as potential modulators of amyloid aggregation [37,38]. Although several seaweed-derived sulfated polysaccharides have been reported to modulate amyloid fibrillation, their effects vary with polysaccharide source, molecular characteristics, and target protein [40,41,42,43,44]. This is particularly significant because polysaccharides can influence protein aggregation through several non-exclusive mechanisms, including macromolecular crowding, electrostatic interactions, and specific interactions with aggregation-prone proteins.

Fucoidan, a heterogeneous sulfated polysaccharide predominantly found in brown algae, is primarily composed of fucose, along with varying amounts of other monosaccharides, uronic acids, acetyl groups, and sulfate groups [45,46]. Its molecular size, monosaccharide composition, sulfation pattern, branching, and glycosidic linkages vary significantly depending on the algal source and preparation method [45,46]. Fucoidans from *Cladosiphon okamuranus*, *Kjellmaniella crassifolia*, *Undaria pinnatifida*, *Sargassum fusiforme*, and *Sargassum horneri* have been studied for their diverse biological activities. This heterogeneity renders it inappropriate to consider “fucoidan” as a single chemically defined substance and complicates direct structure-activity assignments without detailed compositional and structural characterization.

This study investigated the inhibitory effect of *Sargassum horneri* fucoidan (S-fucoidan) on insulin amyloid fibrillation in vitro. Insulin was used as a model amyloid-forming protein to facilitate the analysis of the fibrillation process. Initial screening compared fucoidan preparations derived from *Cladosiphon okamuranus* (C-fucoidan), *Kjellmaniella crassifolia* (K-fucoidan), and *S. horneri* (S-fucoidan), including both high- and hydrolyzed low-molecular-weight preparations, and identified S-fucoidan as the most effective inhibitor. Subsequent analyses focused on S-fucoidan and aimed to (i) determine whether its inhibitory effect persists following molecular weight reduction, (ii) characterize its effects on insulin fibrillation using complementary biophysical and biochemical approaches, (iii) identify the stage of fibrillation at which S-fucoidan is most effective, and (iv) distinguish its inhibitory effect from a general polysaccharide crowding effect. Thus, the present study focused on source-dependent differences among the tested fucoidan preparations, retention of the inhibitory activity of S-fucoidan after hydrolysis, and stage dependence of its anti-fibrillation effect. In particular, time-of-addition experiments distinguished suppression before productive fibril growth from inhibition of fibrillation that was already underway.

## 2. Results

### 2.1. Effects of Fucoidan on Amyloid Fibril Formation of Insulin by ThT Assay

**Figure 1:**
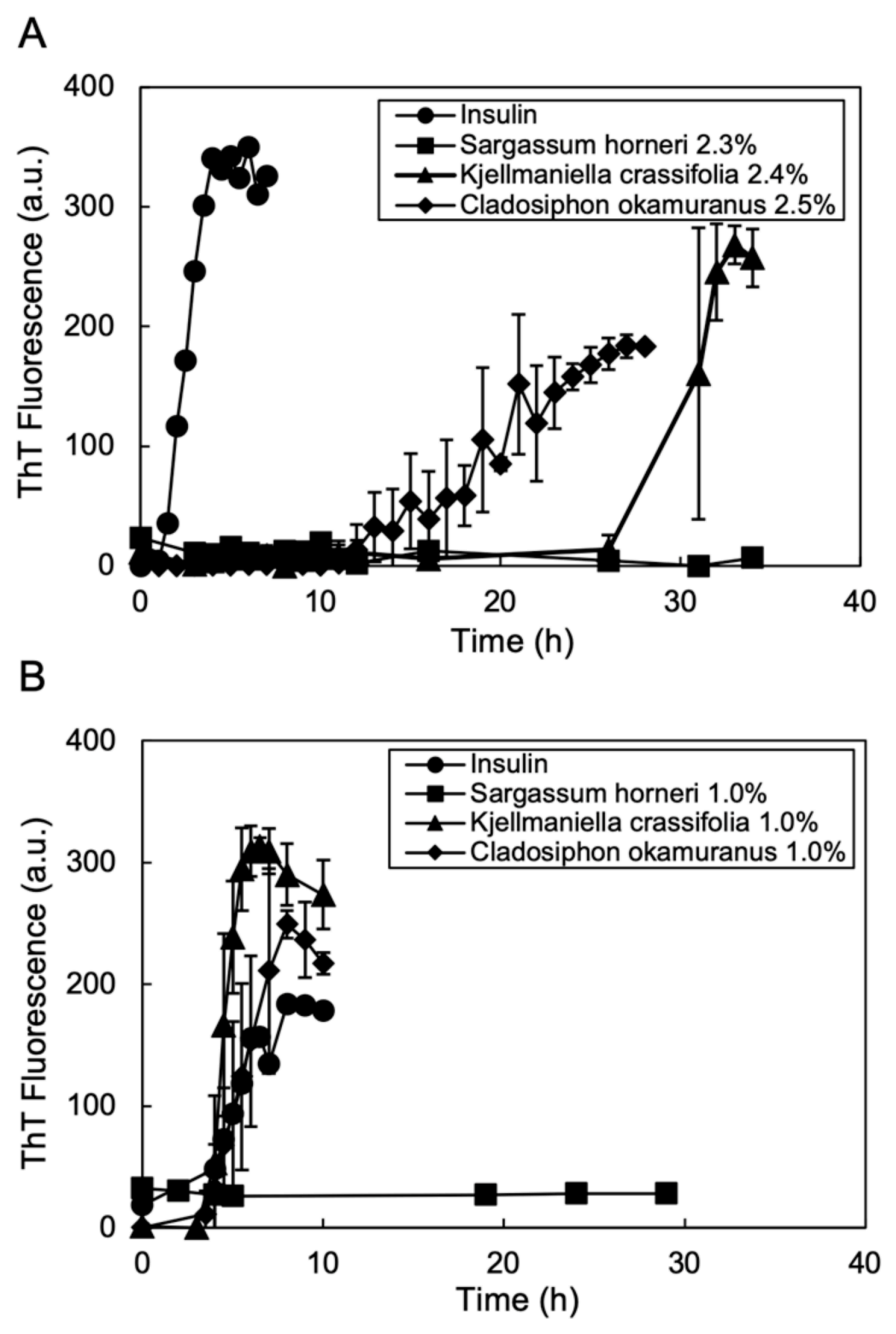
Comparison of the effects of fucoidans from different brown algal sources on insulin fibrillation monitored by ThT fluorescence. (**A**) High-molecular-weight fucoidans from C-fucoidan (2.5% (*w*/*v*): diamond), K-fucoidan (2.4% (*w*/*v*): triangle), and S-fucoidan (2.3% (*w*/*v*): square). (**B**) Hydrolyzed low-molecular-weight fucoidans from C-fucoidan (1.0% (*w*/*v*): diamond), K-fucoidan (1.0% (*w*/*v*): triangle), and S-fucoidan (1.0% (*w*/*v*): square). Insulin without fucoidan was used as a control (circle). Data are presented as mean ± SD of three independent experiments (n = 3).

To examine the effect of fucoidan on insulin fibrillation, three distinct preparations were analyzed: C-fucoidan from *C. okamuranus*, K-fucoidan from *K. crassifolia*, and S-fucoidan from *S. horneri* (Figure 1). In the control reaction, insulin fibrillation commenced after approximately 1.5 h, as evidenced by an increase in ThT fluorescence, and reached a plateau within approximately 8 h (Figure 1A, closed circle). At the initial concentrations tested (2.3–2.5% *w*/*v*), all three fucoidans delayed insulin fibrillation, although the extent of this effect varied among the different fucoidans. Specifically, C-fucoidan and K-fucoidan delayed the increase in ThT fluorescence to approximately 10 and 25 h, respectively, whereas S-fucoidan did not exhibit a detectable increase in ThT fluorescence throughout the measurement period. These findings indicate that S-fucoidan exerted the most pronounced suppressive effect under the tested conditions.

The weight-average molecular weights (Mw), determined via HPLC-SEC using pullulan standards, were approximately 150,000 Da for C-fucoidan, 258,000 Da for K-fucoidan, and 212,000 Da for S-fucoidan, respectively. Given that polysaccharides are polydisperse mixtures, these values represent the apparent Mw rather than distinct molecular masses. To determine whether the high molecular weight and viscosity of fucoidans were necessary for the observed effects, low-molecular-weight (LMw) fucoidans were prepared by hydrolysis. The apparent Mw of these fucoidans were approximately 12,900 Da for LMw-C-fucoidan, 1800 Da for LMw-K-fucoidan, and 6000 Da for LMw-S-fucoidan, respectively. At a concentration of 1.0% (*w*/*v*), LMw-C- and LMw-K-fucoidans did not significantly inhibit insulin fibrillation, whereas LMw-S-fucoidan exhibited a strong inhibitory effect (Figure 1B). Consequently, subsequent analyses focused on S-fucoidan. These comparisons demonstrate source-dependent differences among the tested fucoidan preparations but do not establish a structure–activity relationship.

**Figure 2:**
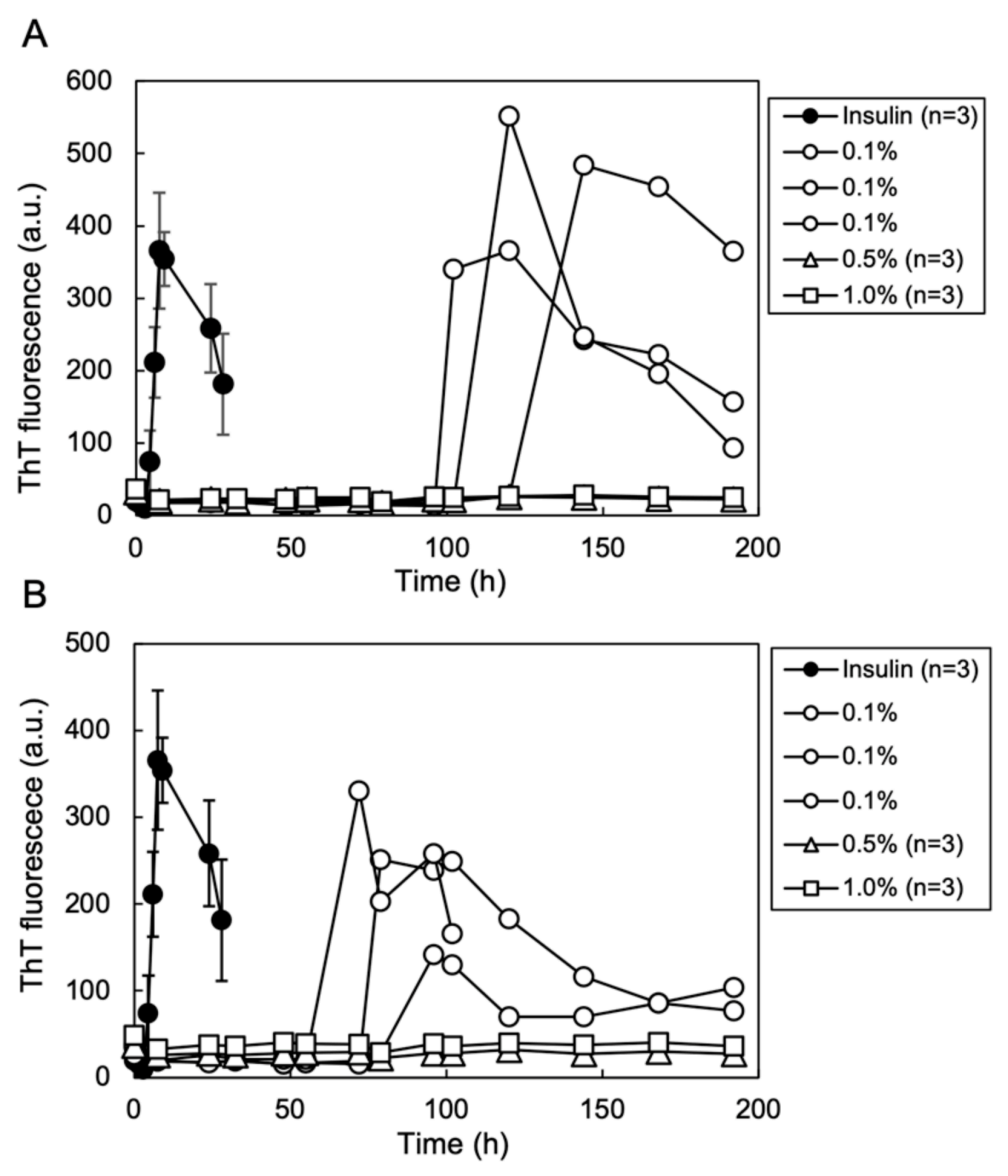
Concentration-dependent effects of S-fucoidan on insulin fibrillation monitored by ThT fluorescence. (**A**) S-fucoidan and (**B**) LMw-S-fucoidan. Insulin alone, closed circles; 0.1% (*w*/*v*) S-fucoidan, open circles; 0.5% (*w*/*v*), open triangles; 1.0% (*w*/*v*), open squares. Three independent experiments were performed for each condition (n = 3), and the individual kinetic traces are displayed rather than an average curve. At 0.5% and 1.0% (*w*/*v*), the three experiments showed little or no detectable increase in ThT fluorescence.

Figure 2 shows the concentration-dependent effects of S-fucoidan (Figure 2A) and LMw-S-fucoidan (Figure 2B) on insulin fibrillation in vitro. At 0.5% and 1.0% (*w*/*v*), S-fucoidan effectively inhibited any detectable increase in ThT fluorescence for approximately 200 h. However, at 0.1% (*w*/*v*), the increase in fluorescence was delayed until approximately 100 h, demonstrating a concentration-dependent effect. Similarly, LMw-S-fucoidan significantly suppressed fibrillation at 0.5% and 1.0% (*w*/*v*), although its effect was markedly diminished at 0.1% (*w*/*v*). Both S-fucoidan and LMw-S-fucoidan exhibited pronounced activity at higher concentrations, whereas lower concentrations highlighted the difference between the two preparations. Because hydrolysis may alter properties other than the apparent molecular weight, these data do not identify molecular weight as the sole determinant of activity.

### 2.2. Morphologies of Insulin Aggregation by Transmission Electron Microscopy (TEM)

**Figure 3:**
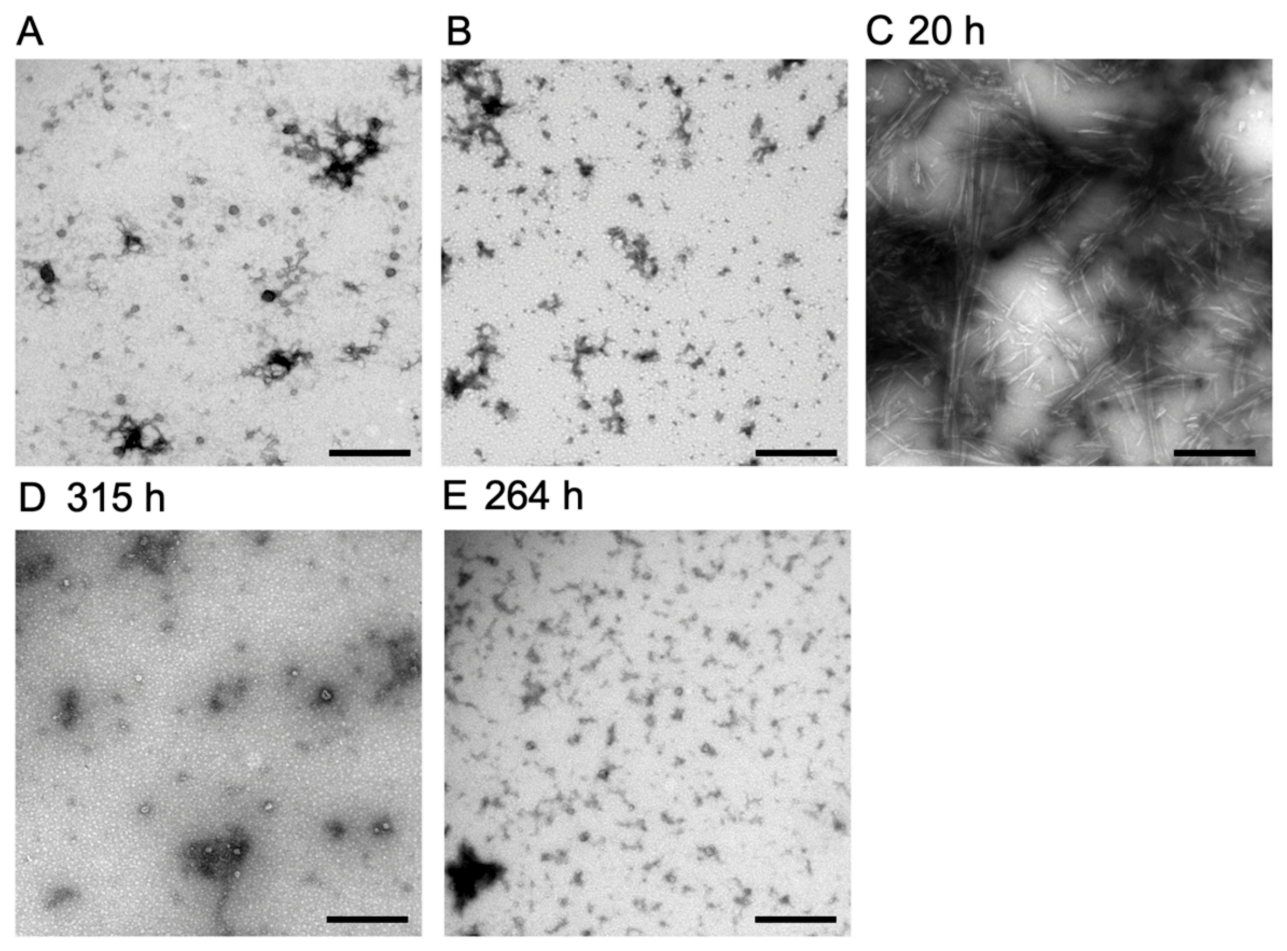
Representative TEM images of samples used to evaluate insulin fibrillation in the presence and absence of S-fucoidan. (**A**) S-fucoidan solution; (**B**) LMw-S-fucoidan solution; (**C**) insulin amyloid fibrils after 20 h; (**D**) insulin incubated with 0.5% (*w*/*v*) S-fucoidan for 315 h; and (**E**) insulin incubated with 0.5% (*w*/*v*) LMw-S-fucoidan for 264 h. Scale bars are 200 nm.

ThT fluorescence may not be detected if binding to fibrils is inhibited by the added additives or if ThT reacts directly with the additives. Therefore, we employed TEM as an orthogonal technique to determine the sample morphology. Numerous non-fibrillar aggregates were observed in solutions containing either S-fucoidan or LMw-S-fucoidan alone, consistent with the self-association of fucoidans (Figure 3A,B). Typical insulin amyloid fibrils were observed after 20 h in the absence of S-fucoidan (Figure 3C). Conversely, no typical amyloid fibrils were detected in insulin samples incubated with S-fucoidan for the extended periods examined; instead, amorphous aggregates were observed (Figure 3D,E). As TEM alone cannot definitively attribute this material to either fucoidan or insulin, these images were interpreted in conjunction with far-UV circular dichroism (CD) spectra and size-exclusion chromatography (SEC) analyses presented below. Collectively, the TEM observations support the conclusion that the reduced ThT signal cannot be attributed solely to interference with ThT detection.

### 2.3. Monitoring of the Secondary Structure of Insulin During the Reaction

**Figure 4:**
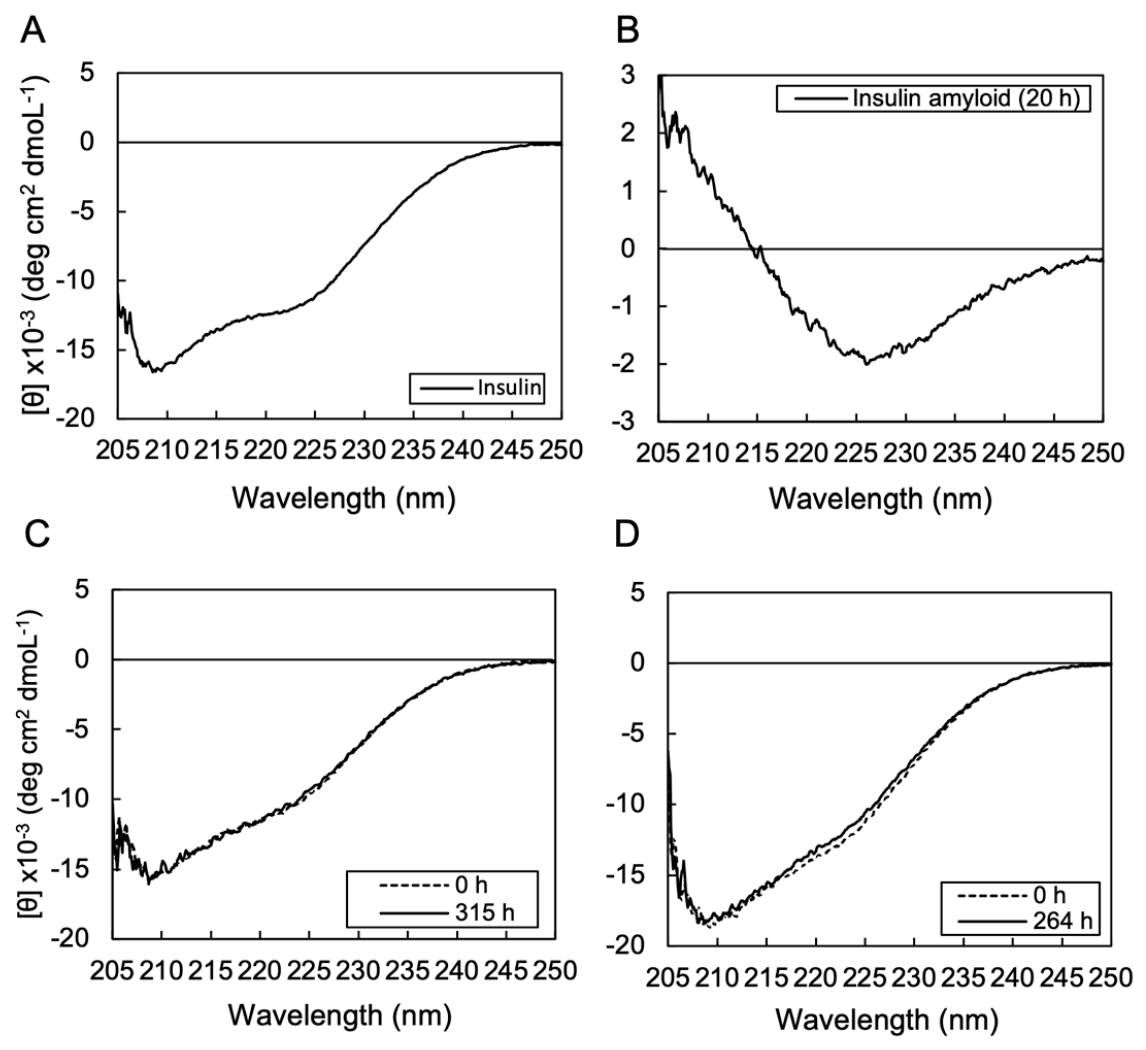
Monitoring the secondary structure of insulin using far-UV CD spectroscopy. (**A**) Insulin before fibrillation. (**B**) Insulin amyloid sample after 20 h of incubation. (**C**,**D**) Insulin incubated with 0.5% (*w*/*v*) S-fucoidan (**C**) and 0.5% (*w*/*v*) LMw-S-fucoidan (**D**), showing spectra before and after prolonged incubation.

TEM revealed that typical insulin amyloid fibrils were absent in the presence of S-fucoidan or LMw-S-fucoidan. CD spectra were measured to examine whether insulin underwent significant secondary-structural transitions (Figure 4). Initially, insulin exhibited minima near 222 and 209 nm, indicating its α-helical secondary structure (Figure 4A). Following fibrillation without S-fucoidan, the spectrum underwent substantial changes, aligning with the characteristic β-sheet-rich state of the insulin amyloids (Figure 4B). In contrast, insulin incubated with S-fucoidan or LMw-S-fucoidan displayed CD spectra similar to those observed before incubation, even after extended reaction periods (Figure 4C,D). These findings suggest that S-fucoidan and LMw-S-fucoidan inhibit extensive secondary-structure transformation associated with insulin amyloid fibrillation under these conditions. The smaller apparent ellipticity amplitude of the fibrillated insulin sample (Figure 4B) should be interpreted cautiously, as amyloid-associated turbidity, light scattering, and/or loss of soluble protein can affect the magnitude and shape of the far-UV CD spectra. Because the concentration of optically accessible insulin in the fibrillated sample was not independently determined, the amplitude difference cannot be assigned specifically to protein precipitation. Therefore, the interpretation relies primarily on the qualitative spectral change, together with TEM and ThT measurements [47].

### 2.4. Detecting Molecular Size of Insulin in the Presence of S-Fucoidan

**Figure 5:**
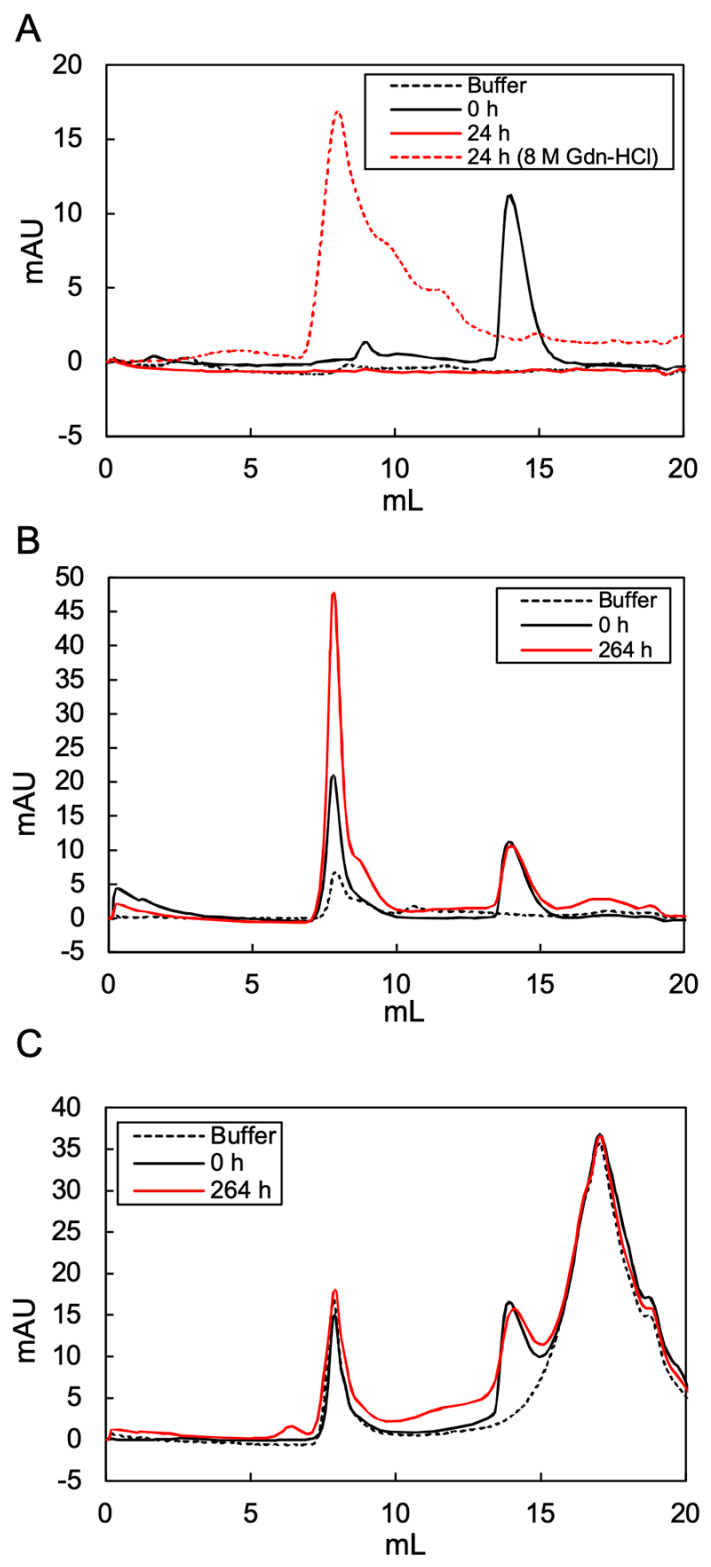
SEC analysis of insulin under various conditions using a Superdex 75 column. (**A**) Amyloid fibrils of insulin. Time 0 h (black line), after 24 h (red line), 8.0 M Gdn-HCl treatment (red dashed line), and reaction buffer (black dashed line). (**B**) Insulin with S-fucoidan at 0 h (black line), 264 h (red line), and reaction buffer, which included S-fucoidan (black dashed line). (**C**) Insulin with LMw-S-fucoidan at 0 h (black line), 264 h (red line), reaction buffer, and including LMw-S-fucoidan (black dashed line).

In the presence of S-fucoidan, insulin maintained its α-helix structure during fibrillation. Additionally, TEM did not detect any amyloid fibrils, leaving the conformational state of insulin unclear. SEC was performed to determine the molecular state of insulin (Figure 5 and Figure S1). Two types of columns were utilized: Superdex 75 (Figure 5), which focused on insulin monomers and oligomers, and Superdex 200 (Figure S1), which targeted larger insulin molecular species. As a control, the insulin monomer eluted at 14 mL on the Superdex 75 column and 18 mL on the Superdex 200 column. Figure 5A and Figure S1A present the results for insulin amyloid fibrils. At 0 h, monomer peaks were observed in both columns (black lines). Insulin amyloid was applied to the SEC columns after 24 or 19 h of incubation, with no signals detected in either column, indicating that the large molecular size of insulin amyloid was trapped in both columns (red line). To solubilize insulin amyloids, 8.0 M Gdn-HCl was applied to both columns, resulting in the elution of insulin with large molecular sizes and no monomer peaks detected (dashed red line). In contrast, insulin samples with S-fucoidan and LMw-S-fucoidan showed notable results. Initially, we checked whether the S-fucoidan and LMw-S-fucoidan solutions displayed peaks at the same elution volume as the insulin monomer (Figure 5B,C, Figure S1B,C, black dashed line). No elution peaks were observed for either of the S-fucoidan solutions. S-fucoidan and LMw-S-fucoidan exhibited insulin monomer peaks after 264 h of incubation (Figure 5B,C, Figure S1B,C, red lines). However, as shown in Figure 5B, although the intensity of the insulin monomers remained unchanged during the reaction, the peak at the void volume increased after 264 h of incubation. Because the insulin monomer position peak was retained, the increase in the void volume may reflect fucoidan self-association or other high-molecular-weight materials. 8.0 M Gdn-HCl was applied to the column (Figure S1B,C, red dashed lines). No peaks were detected, indicating that large aggregates were not formed in the presence of S-fucoidans. Therefore, the unchanged insulin monomer peak intensity before and after the reaction suggests that S-fucoidan and LMw-S-fucoidan suppressed fibrillation while maintaining insulin in a predominantly monomeric or near monomeric state. The amorphous material observed by TEM cannot be definitively assigned to S-fucoidan or insulin, and the present data do not establish whether insulin induces fucoidan self-association.

### 2.5. Stage-Dependent Effect of S-Fucoidan on Insulin Amyloid Fibrillation

**Figure 6:**
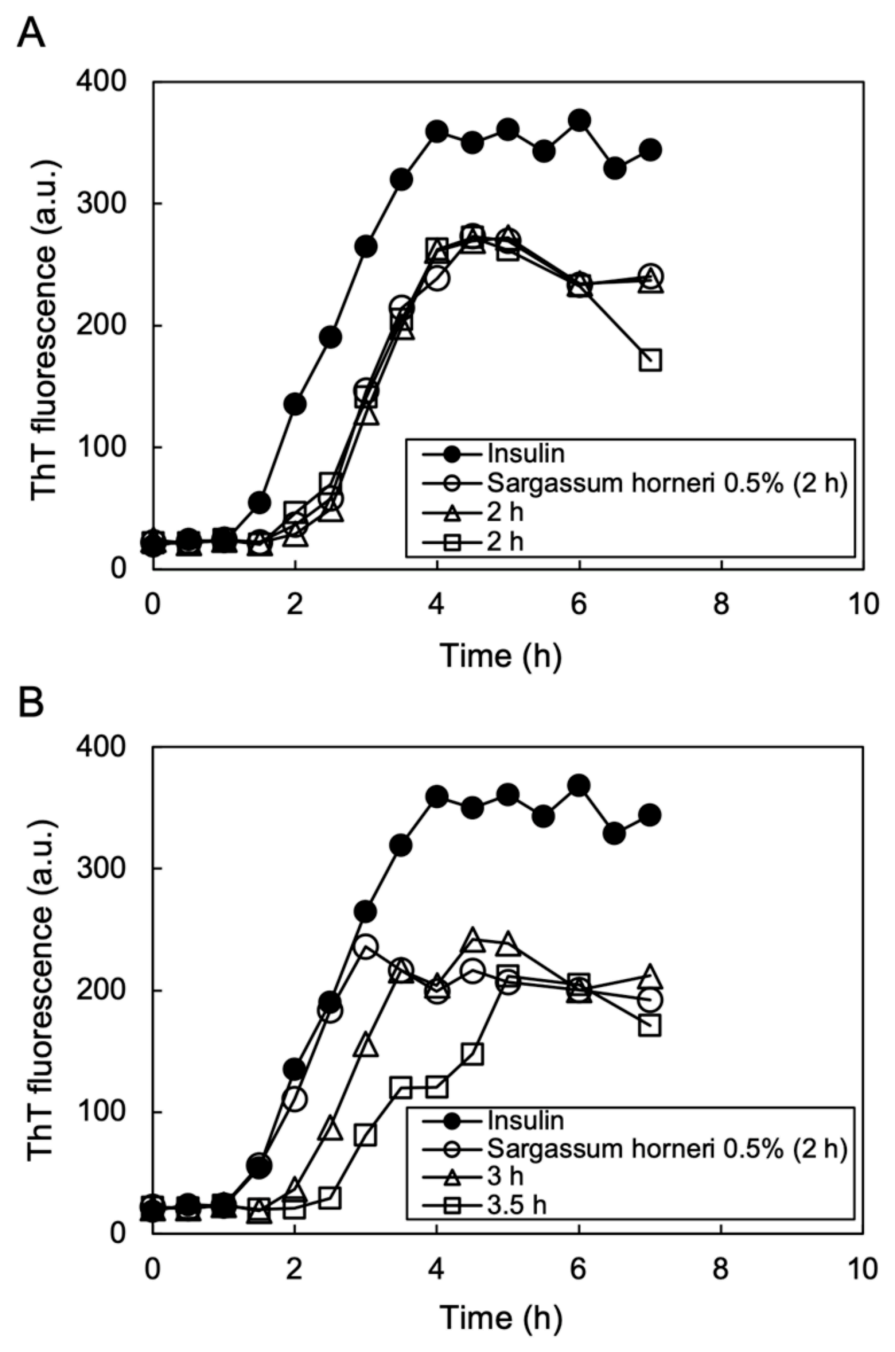
Timing-of-addition analysis of the effect of LMw-S-fucoidan on insulin fibrillation monitored by ThT fluorescence. (**A**) LMw-S-fucoidan was added at 2 h in each of the three independent experiments. (**B**) LMw-S-fucoidan was added when the ThT fluorescence of the corresponding control reaction reached approximately 50% of its plateau level; therefore, the absolute addition times varied slightly among the three experiments. The three independent experiments (n = 3) are distinguished by different open symbols on both panels. Reactions in which LMw-S-fucoidan was present from the start and insulin without fucoidan were included for comparison.

To determine the stage of fibrillation at which S-fucoidan was most effective, LMw-S-fucoidan was added after the initiation of the fibrillation reaction (Figure 6). When 0.5% (*w*/*v*) LMw-S-fucoidan was present from the start of the reaction, the increase in ThT fluorescence was strongly suppressed. In Figure 6A, LMw-S-fucoidan was added at 2 h in each of three independent experiments at an early stage of the ThT increase; the subsequent increase in fluorescence was not prevented. In Figure 6B, the additive was introduced separately in each independent experiment when the corresponding control reaction reached approximately 50% of its eventual ThT fluorescence plateau. Because the fibrillation kinetics differed slightly among experiments, the absolute addition times were not identical. Under this stage-based criterion, addition during the growth phase did not halt the ongoing fluorescence increase. Thus, the inhibitory effects were strongly dependent on the timing of S-fucoidan addition.

### 2.6. Cytotoxicity of Insulin in the Presence of S-Fucoidan

SEC analysis indicated that insulin remained predominantly in a monomeric or near-monomeric state in the presence of S-fucoidan, prompting us to examine whether cytotoxic insulin aggregates were generated under these conditions. For the cytotoxicity assays, insulin was prepared with 1.0% (*w*/*v*) S-fucoidan and incubated for approximately 200 h (Figure S2A). As shown in Figure 2 and Figure 5, both S-fucoidan and LMw-S-fucoidan yielded similar results in terms of insulin fibrillation inhibition. However, Gdn-HCl, which is used in the insulin fibrillation process, may significantly affect cell viability. Ultracentrifugation is required to remove Gdn-HCl; however, separating LMw-S-fucoidan using this method is difficult. Consequently, S-fucoidan, which can be effectively separated, was used in the present study. Initially, the effects of insulin monomers and insulin amyloid fibrils on Neuro-2a cell viability were examined (Figure S2B, white bars). The viability of Neuro-2a cells remained at 80% with various concentrations of insulin monomers, indicating that insulin monomers did not exhibit cytotoxicity under the experimental conditions used in this study. Conversely, the viability of Neuro-2a cells decreased from 67% to 54% depending on the concentration of insulin amyloid, suggesting that insulin is cytotoxic when amyloids are formed (Figure S2B, gray bars). The MTT assay was used as a comparative cytotoxicity assay for insulin samples before and after fibrillation and after incubation with S-fucoidan. Importantly, the material applied to cells after ultracentrifugation was the washed and resuspended precipitated fraction, not the supernatant. Accordingly, the separate SEC observation of a retained monomer-position peak cannot be considered a direct analysis of the MTT test fraction.

No cytotoxicity was detected in Neuro-2a cells exposed to S-fucoidan (Figure S2C). In the MTT assays, precipitated fractions obtained by ultracentrifugation after incubating insulin with S-fucoidan were used to minimize the influence of Gdn-HCl. The control was prepared using an S-fucoidan solution that was incubated for the same duration. Although precipitated insulin amyloid fibrils reduced cell viability, no significant reduction in cell viability was observed in the precipitated fraction obtained from insulin samples incubated with S-fucoidan. Together with the SEC results showing no detectable formation of large insulin assemblies, these findings are consistent with the suppression of cytotoxic insulin-aggregate formation under these conditions. Although amyloidogenic proteins can affect MTT-based estimates of viability, all the test groups were compared using the same assay. S-fucoidan alone did not reduce the MTT-based viability readouts under the examined conditions. Fucoidan also showed no detectable absorbance at the assay wavelength under the conditions tested, reducing concerns about direct optical interference, although this does not exclude all possible assay interference [48].

### 2.7. Comparison with Other Polysaccharides

To examine whether the pronounced suppression observed with LMw-S-fucoidan was also observed with other polysaccharides, insulin fibrillation was monitored in the presence of other hydrolyzed polysaccharides (Figure S3). Under the tested conditions, LMw-starch, LMw-laminaran, LMw-κ-carrageenan, and LMw-λ-carrageenan produced modest delays in fibrillation, whereas 0.5% (*w*/*v*) LMw-S-fucoidan suppressed detectable fibrillation for more than 250 h. Thus, under the conditions examined, the magnitude of the effect observed with LMw-S-fucoidan was not reproduced by the other polysaccharides.

## 3. Discussion

### 3.1. Stage-Dependent Suppression of Insulin Amyloid Fibrillation by S-Fucoidan

Timing-of-addition experiments provide important insights into the stage at which S-fucoidan influences insulin amyloid fibrillation. Although LMw-S-fucoidan strongly suppressed fibrillation when present from the start of the reaction, its addition at the onset of the ThT increase, defined here as the nucleation/transition phase, did not effectively arrest the subsequent fibrillation process (Figure 6). Similarly, addition during the elongation phase failed to stop the ongoing increase in ThT fluorescence. This time-dependence suggests that the pronounced inhibitory effect of S-fucoidan occurs before productive fibril growth is established. Rather than acting primarily as a fibril-end blocker or disaggregating agent, S-fucoidan may interfere with the early molecular events required for insulin to enter a productive amyloid-forming pathway. This distinction is important because compounds that interfere with early assembly can act through mechanisms distinct from those that block fibril elongation or destabilize pre-existing fibrils [30,31]. This interpretation is further supported by the complementary CD, TEM, and SEC results: when S-fucoidan was present from the beginning of the reaction, insulin retained a secondary structure similar to its initial state, typical amyloid fibrils were not detected, and insulin remained predominantly in a monomeric or near-monomeric state. Taken together, these findings support a model in which S-fucoidan predominantly suppresses the early stage of insulin amyloid fibrillation rather than arresting established fibril growth or promoting the removal of pre-existing fibrils.

### 3.2. Comparison with Other Polysaccharides and the Role of Macromolecular Crowding

Macromolecular crowding can either facilitate or inhibit protein aggregation, depending on the specific protein, crowder, and experimental conditions [49]. In the present study, the pronounced suppression observed with LMw-S-fucoidan was not observed with the other polysaccharides examined (Figure S3). These findings suggest that a simple explanation based solely on polymer concentration or nonspecific crowding is insufficient to explain the results. However, owing to variations in the Mw distribution, charge, composition, and solution properties among polysaccharides, the experiments did not identify the specific chemical characteristics of S-fucoidan responsible for these effects. Thus, the comparison should be interpreted as evidence that generic crowding alone does not account for the extent of suppression rather than as definitive proof of a specific molecular interaction.

### 3.3. Working Model and Implications for Sulfated Polysaccharide-Mediated Amyloid Modulation

**Figure 7:**
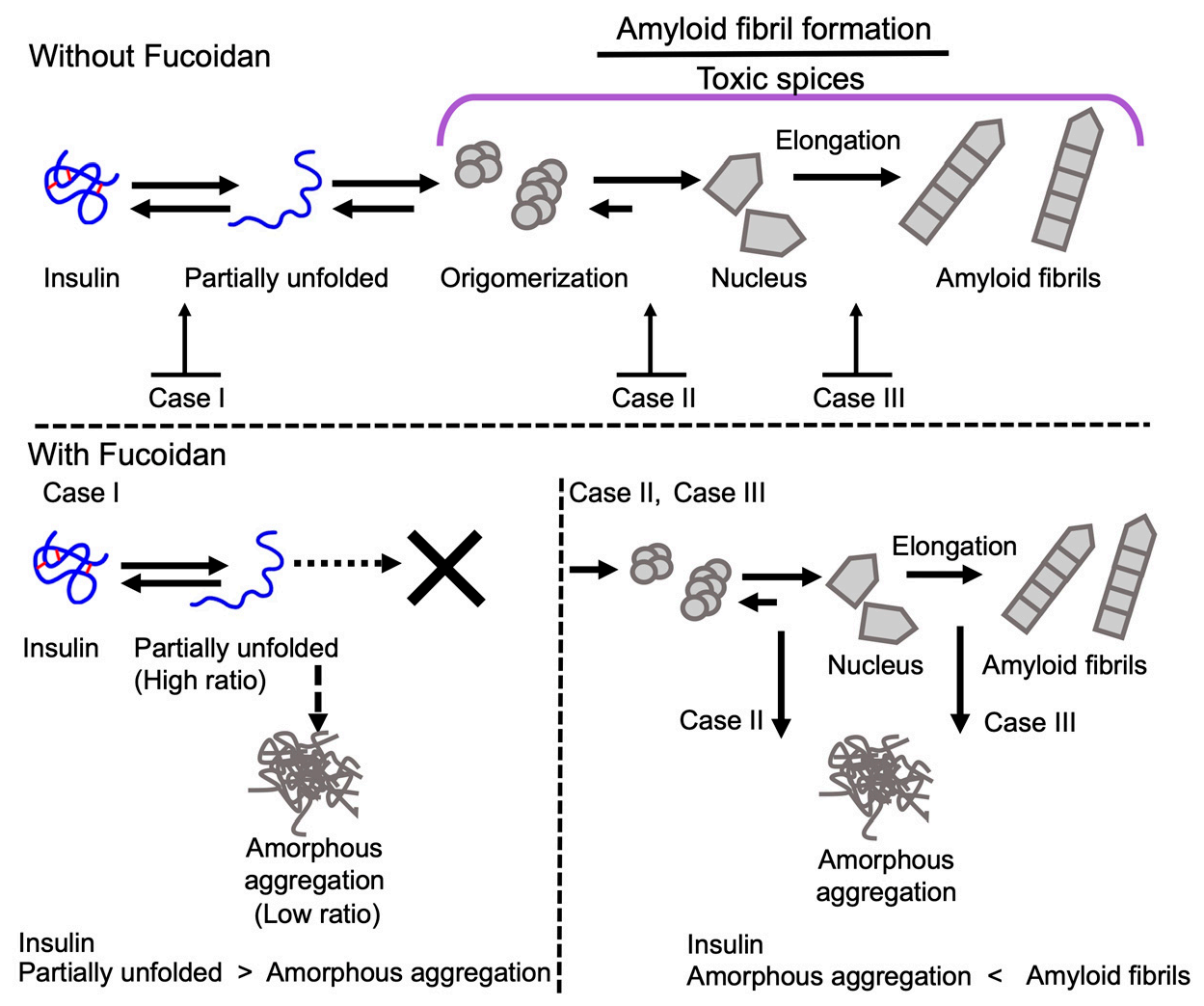
Schematic model of the stage-dependent effect of S-fucoidan on insulin fibrillation. Case I illustrates suppression when S-fucoidan is present before productive fibril growth; Cases II and III illustrate addition during the nucleation/transition and elongation phases, respectively, when ongoing fibrillation is not effectively halted.

Based on these results, we propose a working model in which S-fucoidan interferes with one or more early association events required for insulin to enter a productive fibrillation pathway (Figure 7). In this model, S-fucoidan exerts a pronounced inhibitory effect when present before the establishment of productive fibril growth (Case I), whereas its addition after the onset of nucleation/transition or during elongation does not effectively halt ongoing fibrillation (Cases II and III). However, the molecular interactions underlying this early stage effect remain unclear.

Sulfation alone is unlikely to account for the observed anti-fibrillation activity. Other sulfated polysaccharides, such as heparan sulfate and related glycosaminoglycans, interact with amyloidogenic proteins and can promote or stabilize amyloid assemblies under various conditions [40,50,51,52,53]. In contrast, several algal sulfated polysaccharides have been reported to suppress amyloid formation, suggesting that the effects of sulfated polysaccharides depend on molecular features beyond the mere presence of sulfate groups. This is consistent with prior studies showing that heparin and other glycosaminoglycans can accelerate α-synuclein fibril formation, underscoring that anionic charge alone does not predict the direction of an amyloid-modulating effect [44].

The present study focused primarily on the functional characterization of S-fucoidan rather than establishing a structure–activity relationship among the three fucoidan preparations. The apparent Mw values determined by HPLC-SEC provide an initial physicochemical parameter, while further characterization of monosaccharide composition, sulfate content and position, glycosidic linkages, and molecular-weight distribution will be valuable for identifying the structural features associated with the pronounced activity of S-fucoidan. Importantly, S-fucoidan retained significant inhibitory activity following the hydrolysis procedure used to obtain the LMw preparation. Because hydrolysis may affect physicochemical properties beyond molecular weight, the present results do not isolate molecular size as the determinant of this activity. Nevertheless, the retention of inhibitory activity after hydrolysis provides a useful basis for future structure–function studies. Further structural and interaction analyses should help clarify the molecular features responsible for the pronounced anti-fibrillation activity of S-fucoidan. Published structural studies have illustrated the heterogeneity of fucoidans from *C. okamuranus*, *K. crassifolia*, and *S. horneri*; the literature-based comparison in Supplementary Table S1 describes representative features, not the experimentally established structures of the preparations used here [54,55,56,57,58,59].

The anti-fibrillation activity observed for S-fucoidan is consistent with previous reports showing that algal sulfated polysaccharides can modulate amyloid formation [41,42]. Ascophyllan, a sulfated polysaccharide derived from *Ascophyllum nodosum*, suppresses human insulin amyloid fibrillation and the associated transition from an α-helix-rich to a β-sheet-rich structure [41]. Sulfated polysaccharides from *Chlamydomonas reinhardtii* also inhibited α-synuclein fibrillation and affected ongoing fibrillation and preformed fibrillar structures [42]. Furthermore, studies using carrageenans have demonstrated that structurally different sulfated polysaccharides can exert markedly different effects on amyloid structures, with polysaccharide conformation and charge distribution influencing their effects [43]. Taken together, these observations highlight the diversity of amyloid-modulating activities among sulfated polysaccharides and suggest that their effects are influenced by both the polysaccharide structure and the properties of the amyloid-forming protein. The novelty of the present study lies not in establishing that sulfated polysaccharides can affect amyloid formation, but in demonstrating source-dependent differences among the tested preparations, retention of activity after hydrolysis, and strong timing dependence of S-fucoidan-mediated suppression in the insulin model.

Insulin provides a well-established and experimentally tractable model for investigating the molecular processes underlying amyloid fibrillation [60,61]. Fibrillation involves partial unfolding, subsequent nucleation, and fibril growth, allowing the effects of potential inhibitors on distinct stages of the aggregation pathway to be examined [61]. In this context, the present insulin model enabled us to identify the marked time dependence of S-fucoidan-mediated inhibition and distinguish the suppression of early fibrillation events from the inhibition of established fibril growth. Although amyloid-forming proteins differ substantially in their sequences, structures, and aggregation pathways, the present findings provide a useful basis for examining whether S-fucoidan can similarly modulate the aggregation of disease-associated proteins, including Aβ, tau, α-synuclein, and transthyretin. The fucoidan concentrations used in this study were selected to characterize the anti-fibrillation effect in this in vitro model and should not be directly interpreted as concentrations achievable through dietary intake or systemic exposure to fucoidan.

The choice of a Gdn-HCl-assisted model was informed by the work of Ahmad et al., who systematically examined Gdn-HCl-dependent insulin fibrillation and demonstrated the contributions of insulin conformational changes and, under certain conditions, ionic strength [62]. The 2.0 M Gdn-HCl, 37 °C, shaking conditions were selected as a reproducible, accelerated in vitro fibrillation model for 0.5 mg/mL insulin, not to reproduce the physiological environment. Consequently, the present results cannot establish whether the same degree of inhibition occurs under physiological conditions or in vivo. Oral delivery, particularly for lower-molecular-weight preparations, is a potential future route of administration. However, published intestinal transport and oral absorption studies have focused on fucoidans from other sources and compositions and have not demonstrated the bioavailability or efficacy of the S-fucoidan preparation used in the present study [63,64]. The stability, absorption, distribution, and activity of S-fucoidan at achievable tissue concentrations require separate investigation. Future studies using disease-associated amyloid proteins, detailed structural characterization of the active fucoidan species, and analysis of their molecular interactions will be important for determining the broader applicability and molecular basis of the early stage anti-fibrillation activity of S-fucoidan.

## 4. Materials and Methods

### 4.1. Preparation of Polysaccharides

*Sargassum horneri* (S-fucoidan) and *Cladosiphon okamuranus* (C-fucoidan) fucoidan preparations were provided by Marine Products Kimuraya Co., Ltd. (Sakaiminato, Japan). *Kjellmaniella crassifolia* (K-fucoidan) was obtained from Takara Bio Inc. (Siga, Japan). κ-Carrageenan, λ-carrageenan, and starch were procured from FUJIFILM Wako Pure Chemical Corporation (Tokyo, Japan), and laminaran was acquired from Tokyo Chemical Industry Co., Ltd. (Tokyo, Japan). Low-molecular-weight polysaccharide preparations were produced via autoclave (120 °C, 20 min, 15 psi) hydrolysis.

The apparent weight-average molecular weight (Mw) of each polysaccharide preparation was determined using high-performance liquid chromatography (HPLC) size-exclusion chromatography (Shimadzu Corporation, Kyoto, Japan) with a GMPWXL column (Tosoh Corporation, Tokyo, Japan) and refractive index detection. Calibration was performed using pullulan standards; consequently, the reported values represent pullulan (788,000–5900)-equivalent apparent Mw values and should not be considered as the precise molecular masses of chemically homogeneous species.

Each sample, weighing ten grams, was dissolved in 200 mL distilled water and subjected to dialysis against distilled water for one week prior to sample preparation.

### 4.2. Thioflavin T Assay

To investigate insulin fibrillation, 0.5 mg/mL insulin (Roche, Basel, Switzerland) was dissolved in 20 mM sodium phosphate buffer (NaPi, pH 7.0; FUJIFILM Wako Pure Chemical Corporation, Japan) containing 2.0 M guanidine hydrochloride (Gdn-HCl; Nacalai Tesque, Inc., Japan). The samples (500 µL in 15 mL plastic tube) were incubated at 37 °C with shaking at 105 rpm. In the inhibition experiments, the indicated polysaccharides were present at the start of the reaction, unless otherwise stated: S-fucoidan (0.1–2.3% *w*/*v*), C-fucoidan (1.0–2.5% *w*/*v*), K-fucoidan (1.0–2.4% *w*/*v*), κ-carrageenan (1.0% *w*/*v*), λ-carrageenan (0.7% *w*/*v*), laminaran (2.0% *w*/*v*), and starch (1.0% *w*/*v*). Insulin fibrillation was monitored using ThT fluorescence in an SH-9000 microplate reader (Corona Electric Co., Ltd., Japan). For fluorescence measurements, 150 µL of 10 µM ThT in NaPi and 2 µL of sample were added to a 96-well half-area black plate (Greiner Bio-One, Frickenhausen, Germany). ThT fluorescence was measured at an excitation wavelength of 440 nm and an emission wavelength of 480 nm. This formulation was used to obtain a reproducible in vitro model of insulin fibrillation at the stated protein concentration; the 2.0 M Gdn-HCl condition was not intended to mimic physiological insulin aggregation conditions.

### 4.3. Circular Dichroism Measurement (CD)

The secondary structure of insulin was examined using a Jasco 820 CD spectrophotometer (JASCO Corporation, Tokyo, Japan). Prior to measurement, the samples were diluted with 20 mM NaPi to achieve a concentration range of 0.5–0.1 mg/mL insulin. Far-UV measurements spanning 250–200 nm were conducted at 25 °C using a quartz cuvette with a path length of 1 mm. The results are reported as mean residue ellipticity [θ].

### 4.4. Size Exclusion Chromatography (SEC)

For SEC analysis, a reaction solution (250 µL) was prepared comprising 20 mM NaPi (pH 7.0) and 2.0 M Gdn-HCl, with or without 0.5% (*w*/*v*) S-fucoidan. The samples analyzed included insulin amyloid after a 24-h reaction, samples at 0 h (prior to the initiation of the reaction), and samples incubated for 264 h (post-reaction completion). To assess the molecular size of insulin, the samples were applied to Superdex 75 (molecular weight range: 75,000–3000) and Superdex 200 (molecular weight range: 600,000–10,000) columns, both supplied by Cytiva, USA, using the AKTA pure25 system (Cytiva, Marlborough, MA, USA).

### 4.5. Transmission Electron Microscopy (TEM)

Samples containing amyloid fibrils, with and without S-fucoidan, were deposited onto carbon-coated copper grids (400 mesh; Nissin EM Co., Ltd., Tokyo, Japan). After allowing the sample to stand for one minute, the excess solution was removed using filter paper. The fibrils adsorbed onto the grid were negatively stained with 2% (*w*/*v*) uranyl acetate. Electron micrographs were obtained using a JEM-1400 Plus microscope (JEOL, Tokyo, Japan) operated at an acceleration voltage of 80 kV.

### 4.6. Cell Viability Assay (MTT)

To assess cytotoxicity, MTT assays (CellTiter 96^®^ Non-Radioactive Cell Proliferation Assay, Promega, USA) were performed using mouse Neuro-2a cells obtained from the JCRB Cell Bank (NIBIOHN, Osaka, Japan). Neuro-2a cells were cultured in Gibco Minimum Essential Media (MEM) (Thermo Fisher Scientific, Waltham, MA, USA) supplemented with 10% (*v*/*v*) fetal bovine serum (FBS) (Cosmo Bio Co., Ltd., Tokyo, Japan), 1× MEM non-essential amino acid solution (100×) (FUJIFILM Wako Pure Chemical Corporation, Osaka, Japan), 1 mM sodium pyruvate solution (FUJIFILM Wako Pure Chemical Corporation, Osaka, Japan), and 1% (*v*/*v*) penicillin/streptomycin (Thermo Fisher Scientific, Waltham, MA, USA). The cells were incubated at 37 °C in a 5% CO_2_/95% air atmosphere. Twenty-four hours before the experiment, Neuro-2a cells were seeded onto a 96-well culture plate at a density of 1.0×10^5 cells per well in MEM containing 10% FBS. The MTT assay, which relies on the cleavage of the tetrazolium salt MTT to produce a purple formazan dye by viable cells, was performed according to the manufacturer’s protocol. Cells cultured in 96-well plates were exposed for 24 h to varying concentrations of insulin, insulin fibrils, and insulin combined with 1% S-fucoidan. Prior to the MTT assay, insulin fibrils and insulin with 1% S-fucoidan underwent ultracentrifugation (110,000 × g, 1 h, 4 °C), and each precipitate was washed with sterile, endotoxin-free water (FUJIFILM Wako Pure Chemical Corporation, Osaka, Japan). This ultracentrifugation process was repeated twice, after which the samples were resuspended in sterile, endotoxin-free water for use in the MTT assay. The absorbance of the resulting formazan was measured using a SH-9000 microplate reader at 570 nm (Corona Electric Co., Ltd., Ibaraki, Japan). The mean absorbance of the control wells (cells without insulin) was used as a reference for 100% viability. The viability of cells treated with various insulin formulations was assessed in triplicate and expressed as a percentage of the control cells. The assay was used to compare relative cytotoxic effects under matched conditions rather than to measure cell death directly. S-fucoidan alone was tested as a control, and no detectable absorbance of fucoidan at 570 nm was observed under the conditions examined.

## Data Availability

The original contributions presented in this study are included in the article and the Supplementary Materials. Further inquiries can be directed to the corresponding author.

## Supplementary Materials

The following supporting information can be downloaded at https://www.mdpi.com/article/10.3390/ijms27219516/s1.

## Abbreviations

The following abbreviations are used in this manuscript:

CD	Circular Dichroism
SEC	Size Exclusion Chromatography
TEM	Transmission Electron Microscopy
ThT	Thioflavin T
