# Supplementary material for: Sargassum horneri Fucoidan Suppresses the Early-Stage Amyloid Fibrillation of Insulin
Source: Int J Mol Sci. 2026 Oct 24;27(21):9516. doi: 10.3390/ijms27219516 (PMC13762530; doi:10.3390/ijms27219516)
Supplement: Supplementary file 1 [file ijms-27-09516-s001.zip › ijms-4612508-supplementary.pdf]

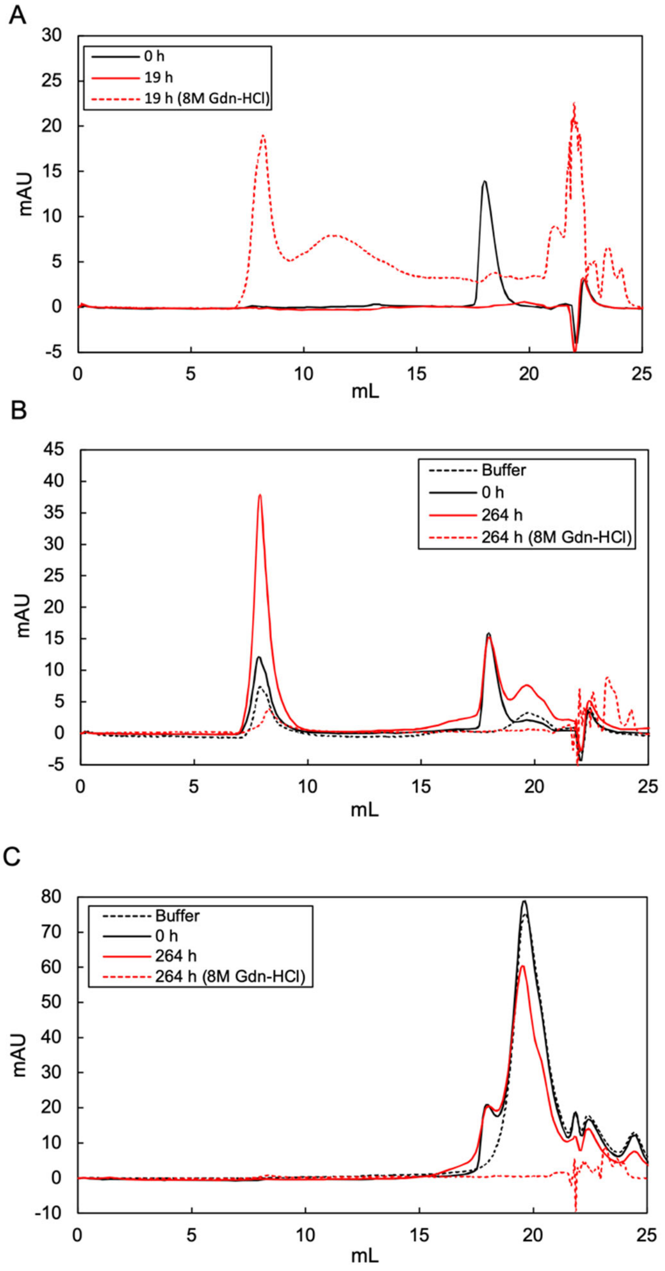

Figure S1. SEC analyses (Superdex 200) of insulin under different conditions. (A) Amyloid fibrils of insulin. Time 0 h (black line), after 24 h (red line), 8.0 M Gdn-HCl treatment (red dashed line), and reaction buffer (black dashed line). (B) Insulin with high molecular weight S-fucoidan at 0 h (black line), after 264 h (red line), 8.0 M Gdn-HCl treatment (red dashed line), and in the reaction buffer including high molecular weight S-fucoidan (black dashed line). (C) Insulin with low molecular weight S-fucoidan at 0 h (black line), 8.0 M Gdn-HCl treatment (red dashed line), and in the reaction buffer including low molecular weight S-fucoidan (black dashed line).

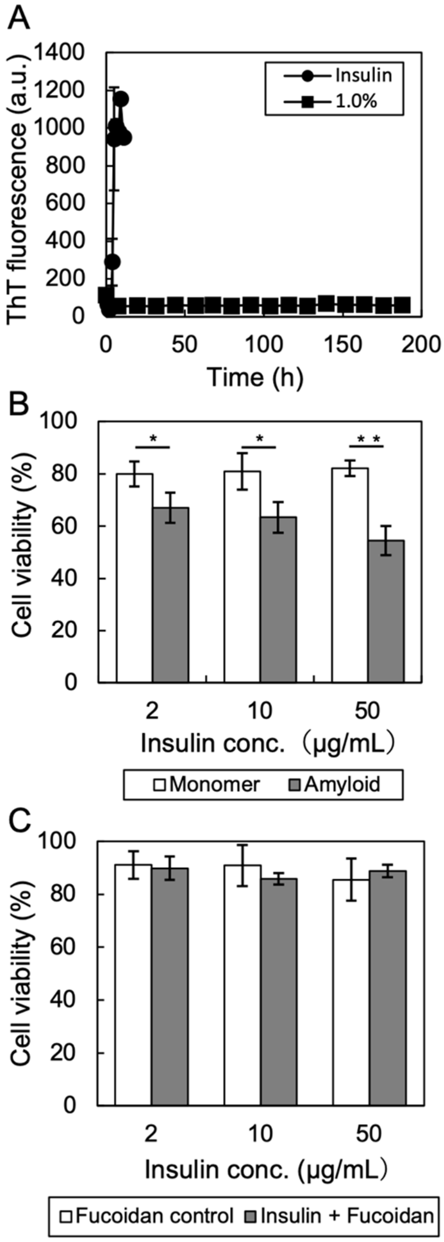

Figure S2. Cytotoxicity of insulin fibrils and insulin with S-fucoidan. (A) Sample preparation of insulin fibrils and insulin with 1.0% S-fucoidan monitored by the ThT assay. Insulin fibrils (closed circles) and insulin with 1.0% S-fucoidan (closed squares). (B) Cell viability of Neuro-2a cells in the presence of insulin monomer (white: n=4) and amyloid fibrils after approximately 10 h of reaction (gray: n=3). (C) Cell viability of Neuro-2a cells in the presence of S-fucoidan (white: n=3) and insulin with S-fucoidan after approximately 190 h of reaction (gray: n = 3). *$p < 0.05$, **$p < 0.01$

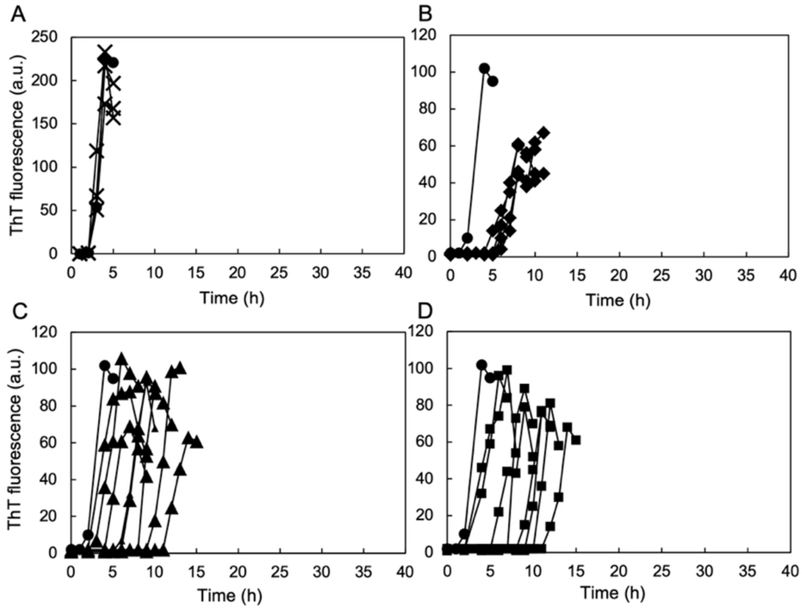

Figure S3. Effects of various polysaccharides on insulin amyloid fibril formation. (A) Starch (cross) n=3 (B) Laminaran (diamond) n=3. (C) λ-carrageenan (triangle) n=8. (D) κ-carrageenan (square) n=8. Control of insulin fibrillation (circle).

Table S1. Literature-reported structural characteristics of fucoidans from three brown algal species.

Structural feature	*Cladosiphon okamuranus*	*Kjellmaniella crassifolia*	*Sagassum horneri*
Major monosaccharides	Fucose and glucuronic acid	Predominantly fucose; galactose is also present	Predominantly fucose
Other reported monosaccharides	Xylose, galactose, mannose, glucose, arabinose, and rhamnose	Mannose and glucuronic acid	Not specified in the cited structural study
Glycosidic linkages	Predominantly α-(1→3)-linked fucose backbone	Predominantly (1→3)-linked fucose residues have been identified in oligosaccharide fragments; heterogeneous galactofucan structures are also reported	α-(1→3)- and α-(1→4)-linked fucose residues
Sulfation pattern	Mainly 4-O-sulfated fucose residues	2-O- and/or 4-O-sulfated fucose residues have been identified in oligosaccharide fragments	Mainly 2-O-sulfated and 2,3-di-O-sulfated fucose residues, with additional 2,4-di-O-sulfated residues
O-Acetyl groups	O-Acetylation of some fucose residues has been reported	O-Acetylated galactofucan fractions have been reported	Not established in the cited structural study
Branching / side chains	Glucuronic acid substitution at C-2 and fucose branching at C-2 have been reported in different studies	Structural heterogeneity among fractions; detailed branching patterns are not uniformly established	Unsulfated fucose-containing side chains attached at C-4 of the main-chain residues
Representative structural characteristics	Sulfated α-(1→3)-L-fucan with glucuronic acid substitution and/or fucose branching	Heterogeneous sulfated fucans and galactofucans	Highly sulfated, branched fucan containing α-(1→3) and α-(1→4) linkages
References	1. Nagaoka et al. (1999)<br>2. Lim et al. (2019)	3. Zhang et al. (2015)<br>4. Song et al. (2018)	5. Silchenko et al. (2017)

Note: The structural characteristics summarized in this table are based on previously published studies of fucoidans isolated from the corresponding brown algal species. Fucoidans exhibit considerable structural heterogeneity depending on the algal source, extraction conditions, and fractionation procedures. In particular, the structural features reported for *Kjellmaniella crassifolia* include findings obtained from analyses of isolated oligosaccharide fragments and different polysaccharide fractions. Therefore, the characteristics listed here should be regarded as representative literature-reported features and do not necessarily reflect the exact chemical structures of the fucoidan preparations used in the present study. The cited studies correspond to References 54–59 in the main article, respectively. Detailed structural characterization of these preparations is required to establish relationships between their chemical structures and anti-fibrillation activities.

References

1. Nagaoka, M.; Shibata, H.; Kimura-Takagi, I.; Hashimoto, S.; Kimura, K.; Makino, T.; Aiyama, R.; Ueyama, S.; Yokokura, T. Structural study of fucoidan from Cladosiphon okamuranus TOKIDA. Glycoconj. J. 1999, 16, 19–26.

2. Lim, S.J.; Wan Aida, W.M.; Schiehser, S.; Rosenau, T.; Böhmdorfer, S. Structural elucidation of fucoidan from *Cladosiphon okamuranus* (Okinawa mozuku). *Food Chem.* **2019**, *272*, 222–226.
3. Zhang, W.; Jin, W.; Sun, D.; Zhao, L.; Wang, J.; Duan, D.; Zhang, Q. Structural analysis and anti-complement activity of polysaccharides from Kjellmaniella crsaaifolia. Mar. Drugs 2015, 13, 1360–1374.
4. Song et al. Structural characterization and antitumor effects of fucoidans from brown algae Kjellmaniella crassifolia farmed in northern China. Int. J. Biol. Macromol. 2018, 119, 125–133.
5. Silchenko et al. Structure, enzymatic transformation, anticancer activity of fucoidan and sulphated fucooligosaccharides from Sargassum horneri. Carbohydr. Polym. 2017, 175, 654–660.
